# Synthesis, crystal structure and Hirshfeld surface analysis of 2-({5-[(naphthalen-1-yl)meth­yl]-4-phenyl-4*H*-1,2,4-triazol-3-yl}sulfan­yl)-1-(4-nitro­phen­yl)ethanone

**DOI:** 10.1107/S2056989024000859

**Published:** 2024-01-26

**Authors:** Trong Duc Le, Tien Cong Nguyen, Thi Kim Dung Hoang, Minh Khoi Huynh, Quang Thang Phan, Luc Van Meervelt

**Affiliations:** a Graduate University of Science and Technology, Vietnam Academy of Science and Technology, 18 Hoang Quoc Viet Street, Cau Giay District, Hanoi, Vietnam; b Institute of Chemical Technology, Vietnam Academy of Science and Technology, 1A Thanh Loc 29 Street, District 12, Ho Chi Minh City, Vietnam; c Faculty of Chemistry, Ho Chi Minh City University of Education, 280 An Duong Vuong Street, District No. 5, Ho Chi Minh City, Vietnam; d Hau Nghia High School, 825 Street Section A, Duc Hoa District, Long An Province, Vietnam; e Department of Chemistry, KU Leuven, Biomolecular Architecture, Celestijnenlaan 200F, Leuven (Heverlee), B-3001, Belgium; University of Durham, United Kingdom

**Keywords:** crystal structure, 1,2,4-triazole, Hirshfeld surface

## Abstract

The title compound, C_27_H_20_N_4_O_3_S, crystallizes in the monoclinic system, space group *P*2_1_/*n*, with *Z* = 4. The global shape of the mol­ecule is determined by the orientation of the substituents on the central 4*H*-1,2,4-triazole ring. The nitro­phenyl ring, phenyl ring, and naphthalene ring system are oriented at dihedral angles of 82.95 (17), 77.14 (18) and 89.46 (15)°, respectively, with respect to the triazole ring. The crystal packing features chain formation in the *b*-axis direction by S⋯O inter­actions. A Hirshfeld surface analysis indicates that the highest contributions to surface contacts arise from contacts in which H atoms are involved.

## Abstract 2

In the title compound, C_27_H_20_N_4_O_3_S, the three aromatic rings are oriented almost perpendicular to the plane of the central 1,2,4-triazole ring.

## 1. Chemical context

Heterocyclic compounds featuring triazole ring systems, particularly 1,2,4-triazole, have gained significant attention in synthetic chemistry due to their versatile applications in medicinal, bioorganic, and industrial contexts. The unique 1,2,4-triazole structure is evident in modern drugs such as fluconazole, voriconazole, itraconazole (anti­fungals), alprazolam (anti-convulsant), and ribavirin (anti­viral) (Amjad *et al.*, 2023 ▸). Furthermore, derivatives incorporating the 1,2,4-triazole moiety are acknowledged for a range of biological activities, including anti­bacterial (Chen *et al.*, 2000 ▸), anti­spasmodic (Balabadra *et al.*, 2017 ▸), anti­diabetic (Wang *et al.*, 2017 ▸; Jabeen *et al.*, 2014 ▸), anti­malarial (Gujjar *et al.*, 2009 ▸), anti­viral (Al-Soud *et al.*, 2004 ▸), and anti­fungal (Lass-Flörl, 2011 ▸) properties. Some compounds derived from 1,2,4-triazole also demonstrate moderate to substantial effects as anti­proliferative (Masood-ur-Rahman *et al.*, 2017 ▸), anti­oxidant (Karrouchi *et al.*, 2016 ▸), and anti­cancer agents (Huang *et al.*, 2017 ▸).

In addition to their bioactivities, naphthalene derivatives are recognized for their anti­microbial, anti­cancer (Salahuddin *et al.*, 2014 ▸), anti-inflammatory (Kaushik *et al.*, 2012 ▸), and anti-depressant (Kumar *et al.*, 2018 ▸) properties. Given the diverse bioactivities associated with both 1,2,4-triazole and naphthalene, we embarked on synthesizing a compound containing both moieties through the *S_N_* 2 reaction. Herein we report the crystal structure and Hirshfeld surface analysis of the title compound, C_27_H_20_N_4_O_3_S, obtained during our efforts to synthesize new compounds that contain a 4-phenyl-4*H*-1,2,4-triazole unit. 
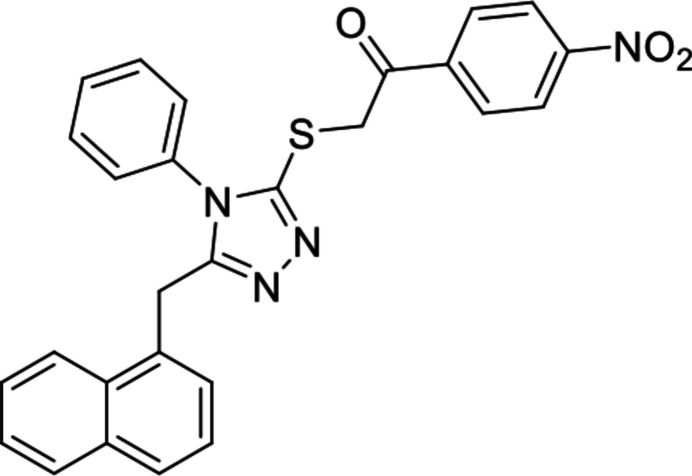


## 2. Structural commentary

**Figure 1:**
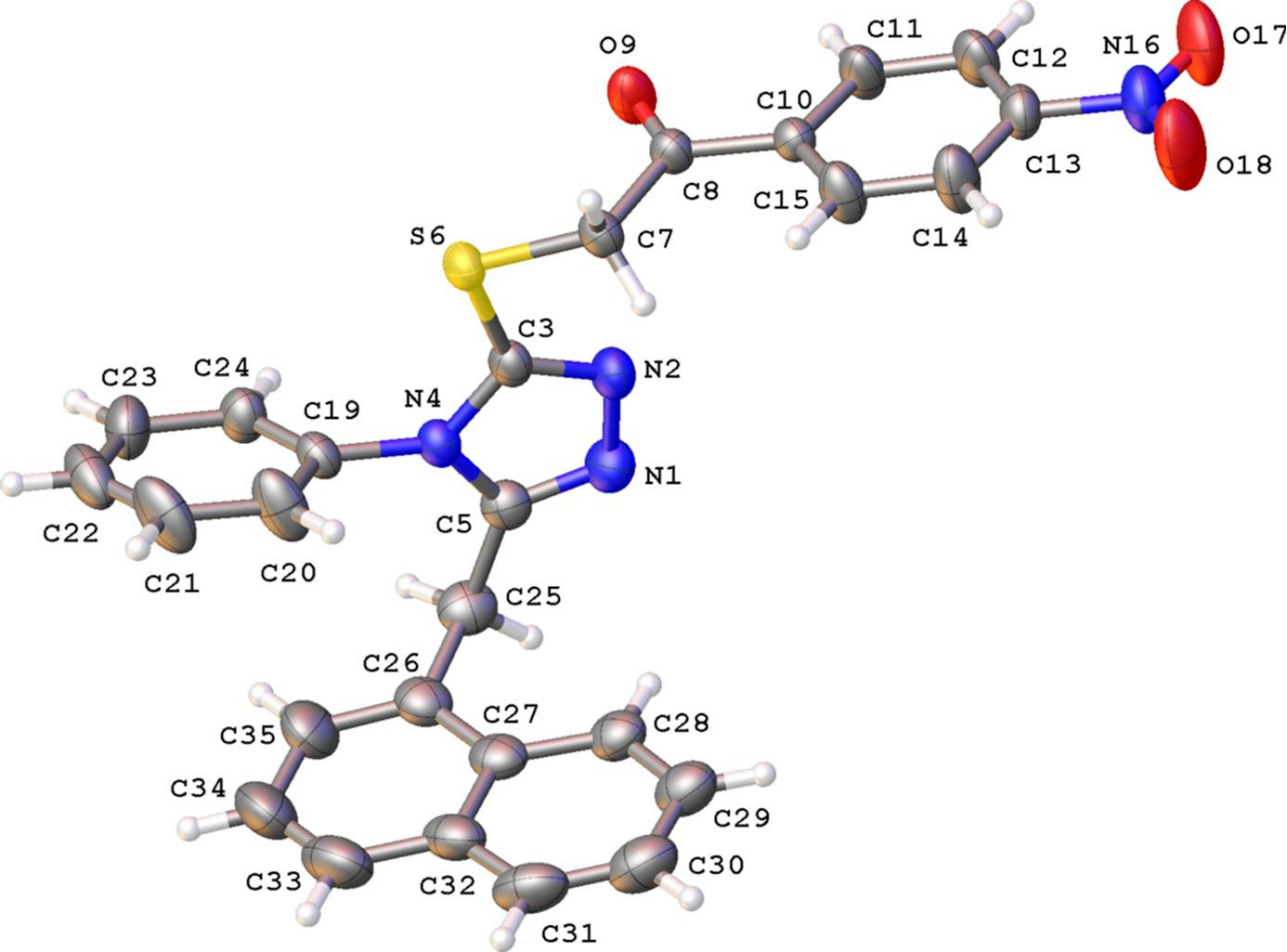
A view of the mol­ecular structure of the title compound, with atom labels and displacement ellipsoids drawn at the 30% probability level. H atoms are shown as small circles of arbitrary radii.

The title compound crystallizes in the monoclinic space group *P*2_1_/*n* with one mol­ecule in the asymmetric unit (Fig. 1 ▸). The central 1,2,4-triazole ring is planar (r.m.s. deviation = 0.002 Å). The three other aromatic rings are oriented almost perpendicular to the plane of the central 1,2,4-triazole ring. The dihedral angles between the 1,2,4-triazole ring and phenyl ring C19–C24, naphthalene moiety C26–C35, and phenyl ring C10–C15 are 77.14 (18), 89.46 (15) and 82.95 (17)°, respectively. The substituent at C3, –SCH_2_C(=O)-nitro­phenyl, is almost planar [r.m.s. deviation = 0.117 Å, largest deviation is 0.301 (1) Å for S6].

## 3. Supra­molecular features

**Figure 2:**
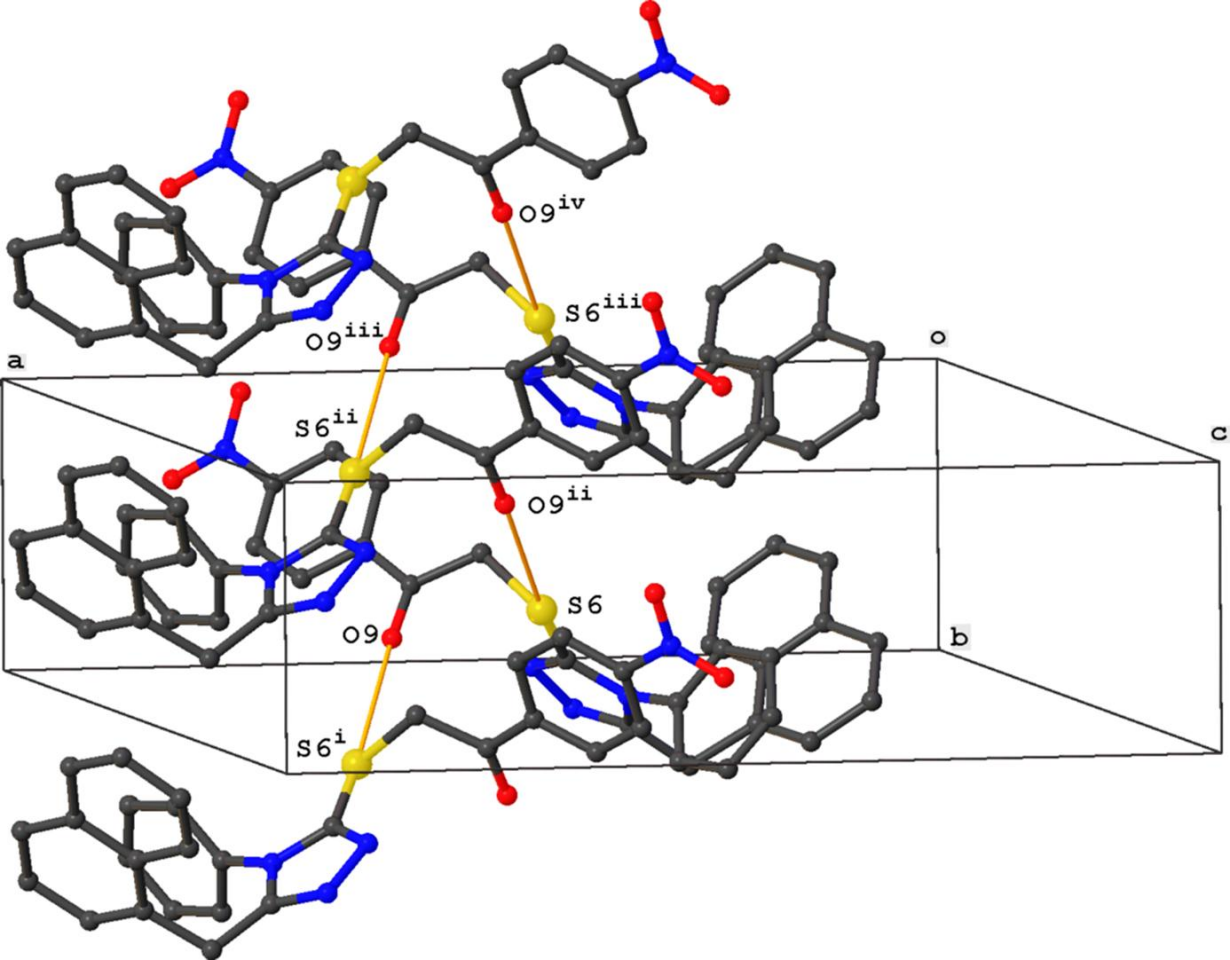
Partial crystal packing of the title compound, showing the chain formation in the *b*-axis direction. S⋯O inter­actions are shown as orange dashed lines. Symmetry codes: (i) −*x* + 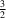 , *y* + 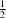 , −*z* + 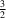 ; (ii) −*x* + 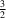 , *y* − 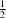 , −*z* + 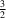 ; (iii) *x*, *y* − 1, *z;* (iv) −*x* + 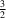 , *y* − 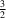 , −*z* + 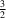 .

**Figure 3:**
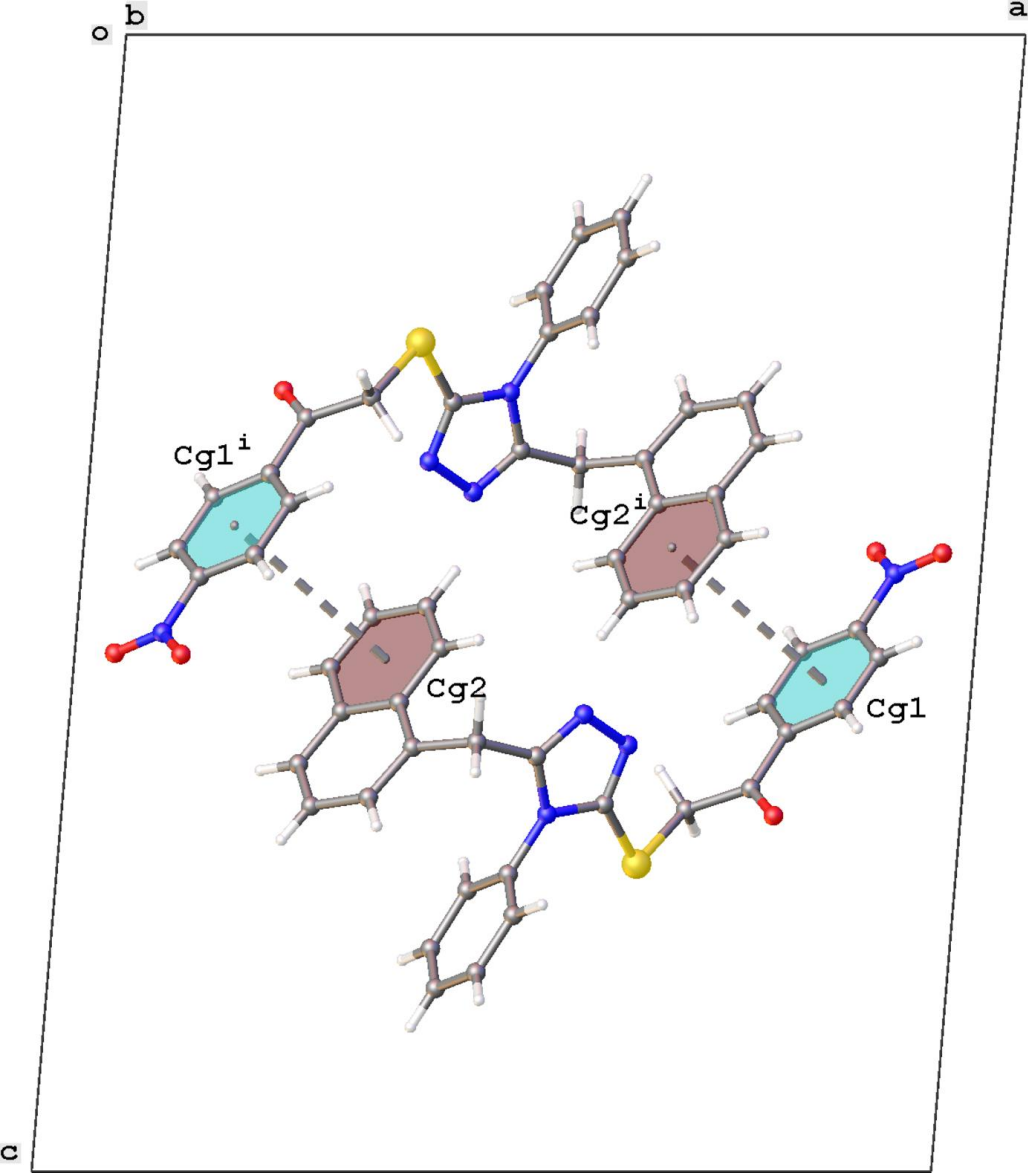
Partial crystal packing of the title compound, showing the π–π stacking. *Cg*1 and *Cg*2 are the centroids of rings C10–C15 and C27–C32, respectively. Symmetry code: (i) −*x* + 1, −*y* + 1, −*z* + 1.

The crystal packing of the title compound is characterized by S⋯O inter­actions between neighboring mol­ecules [O9⋯S6^i^ = 3.115 (3) Å; S6⋯O9^ii^ = 3.115 (3) Å; symmetry codes: (i) −*x* + 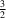 , *y* + 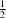 , −*z* + 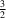 ; (ii) −*x* + 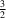 , *y* − 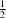 , −*z* + 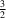 ], resulting in the formation of chains with a *C*(4) graph-set motif running in the *b*-axis direction (Fig. 2 ▸). No classical hydrogen bonds are observed. Despite the presence of multiple aromatic rings, the packing shows no strong π–π or C—H⋯π inter­actions. The shortest distance between aromatic rings is observed for rings C10–C15 and C27–C32, resulting in the formation of inversion dimers. The centroid–centroid distance is 4.105 (2) Å, the dihedral angle between the planes is 6.39 (18)°, and the slippage is 1.708 Å (Fig. 3 ▸).

**Figure 4:**
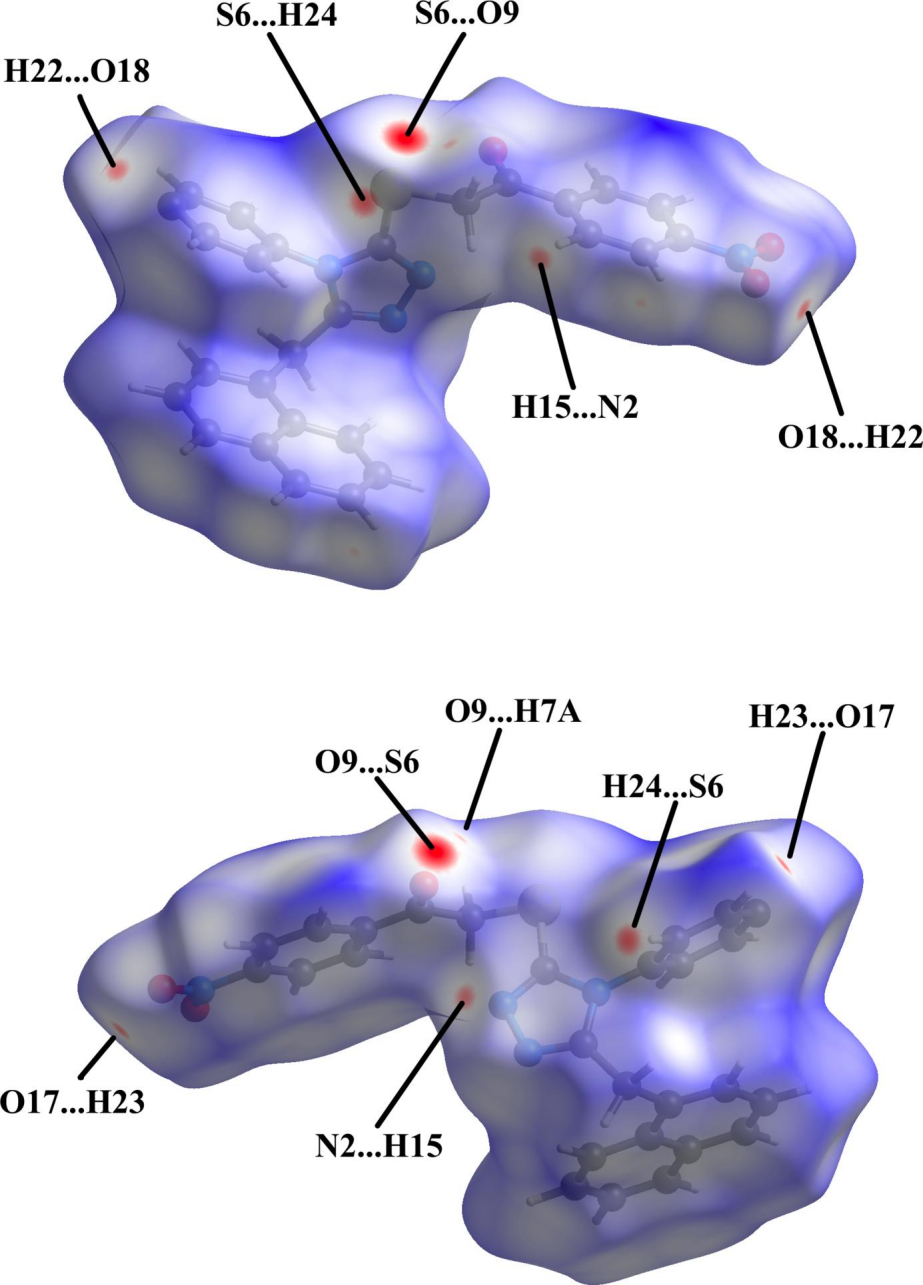
Views of the three-dimensional Hirshfeld surface of the title compound plotted over *d*_norm_ in the range −0.1286 to 1.6073 a.u.

To visualize the inter­molecular inter­actions in the crystal packing in more detail, a Hirshfeld surface (HS) analysis (Hirshfeld, 1977 ▸) was carried out with *Crystal Explorer 21.3* (Spackman *et al.*, 2021 ▸). In the HS plotted over *d*_norm_ (Fig. 4 ▸), a number of short contacts (shorter than the sum of the van der Waals’ radii) are visible as red spots. Further details are given in Table 1 ▸.

**Figure 5:**
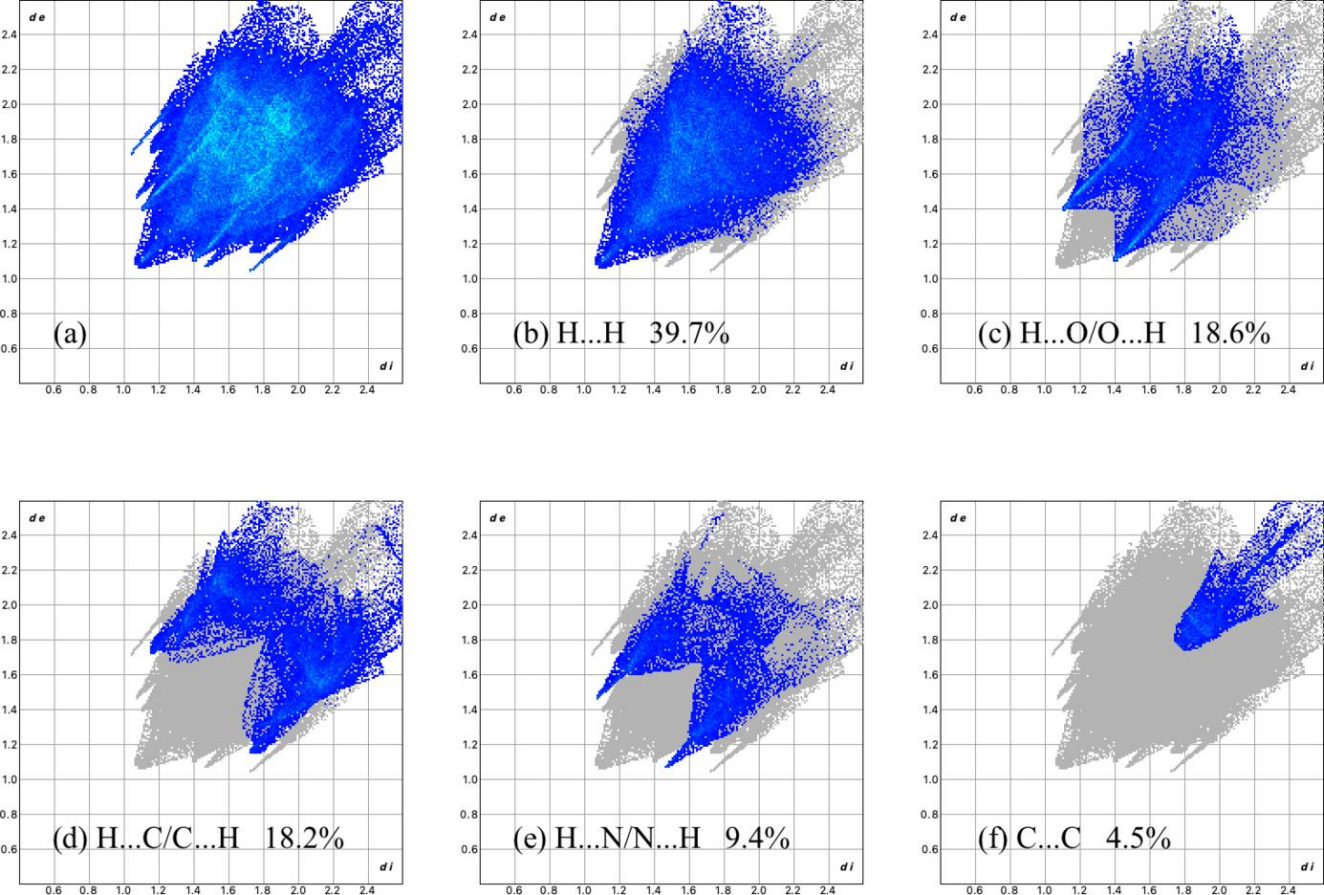
The full two-dimensional fingerprint plots for the title compound, showing (*a*) all inter­actions, and delineated into (*b*) H⋯H, (*c*) H⋯O/O⋯H, (*d*) H⋯C/ C⋯H, (*e*) H⋯N/N⋯H, and (*f*) C⋯C inter­actions. The *d*_i_ and *d*_e_ values are the closest inter­nal and external distances (in Å) from given points on the Hirshfeld surface.

The overall two-dimensional fingerprint plot, Fig. 5 ▸ *a*, and those delineated into H⋯H, H⋯O/ O⋯H, H⋯C/C⋯H, H⋯N/N⋯H and C⋯C contacts (McKinnon *et al.*, 2007 ▸) are illustrated in Fig. 5 ▸ *b*–*f*, respectively, together with their relative contributions to the Hirshfeld surface. The pairs of spikes with tips at *d_e_* + *d_i_* = 2.55 Å in Fig. 5 ▸ *c* and Fig. 5 ▸ *e* indicate weak hydrogen-bonding inter­actions. The most significant contributions to the Hirshfeld surface are H⋯H (39.7%), H⋯O/O⋯H (18.6%), H⋯C/C⋯H (18.2%), and H⋯N/N⋯H (9.4%), indicating that the highest contributions arise from contacts in which H atoms are involved. Except for C⋯C (4.5%), the other contributions are less than 2.0%.

## 4. Database survey

A search of the Cambridge Structural Database (CSD, Version 5.44, update of September 2023; Groom *et al.*, 2016 ▸) for the 4-phenyl-4*H*-1,2,4-triazol-3-yl­thio fragment resulted in 70 hits (for refcodes, see supporting information). All 1,2,4-triazole rings are planar (maximum deviation from planarity is 0.010 Å), with the sulfur atom being nearly in the same plane (maximum deviation of 0.163 Å). The dihedral angle between the best planes through the triazole and phenyl ring shows a roughly uniform distribution between 52 and 90°. For the title compound this angle is 77.14 (18)°.

YIBXIU, YIBXEQ and YIBXAM (Le *et al.*, 2023 ▸) are the closest analogues of the title compound, instead of the nitro­phenyl group containing C(=O)NH*R*, where *R* = Ph, *p*-C_6_H_4_-NO_2_ and *p*-tolyl, respectively. The dihedral angles between the triazole ring and its phenyl substituent are 79.96 (15)° for YIBXIU, 66.63 (16), 64.66 (15) and 69.64 (17)° for YIBXEQ (*Z*′ = 3), and 58.29 (9)° for YIBXAM. The packing here is determined by N—H⋯N hydrogen bonds between the amide N—H and one of the triazole nitro­gen atoms.

## 5. Synthesis and crystallization

**Figure 6:**
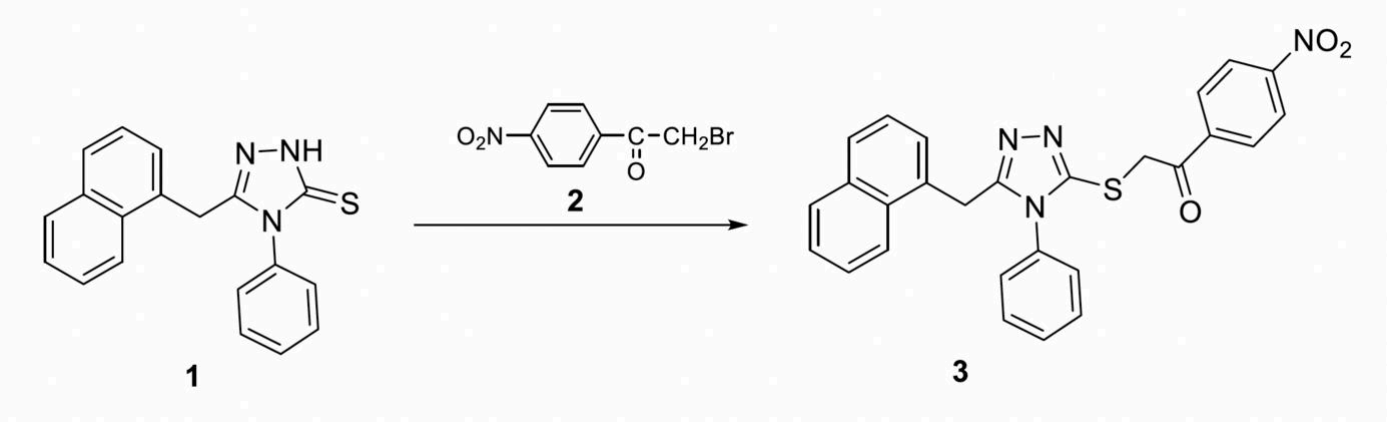
Reaction scheme for the synthesis of the title compound. Compound **1** was identified as the thione by X-ray crystallography, although IR spectra indicate coexistence of the thione and thiol forms in solution (Le *et al.*, 2023 ▸).

The reaction scheme for the synthesis of the title compound is illustrated in Fig. 6 ▸.

5-(Naphthalen-1-ylmeth­yl)-4-phenyl-4*H*-1,2,4-triazole-3-thiol/thione **1** was synthesized through a three-step process as described by Le *et al.* (2023 ▸). 1.0 mmol of compound **1** (0.317 g) was dissolved in ethanol along with 1.0 mmol of 2-bromo-1-(4-nitro­phen­yl)ethanone **2** (0.243 g) and 1.0 mmol of sodium acetate (0.082 g). The reaction mixture was refluxed for 5 h, and upon cooling, it was poured into ice–water. The resulting solid was filtered off and recrystallized from a 1:1 mixture of ethanol and water to give the title compound **3** as plate-like yellow crystals (yield: 76.8%, m.p: 454.5 K).

The IR spectrum for the title compound was recorded using a Shimadzu FT-IR Affinity-1S spectrometer. ^1^H-NMR (500 MHz) and ^13^C-NMR (125 MHz) spectra were obtained utilizing a Bruker Advance spectrometer, with DMSO-*d_6_* serving as the inter­nal standard and solvent. Mass spectra were generated using a Bruker microTOF-Q 10187 instrument. IR (ν, cm^−1^): 3111, 3048 (C-H aromatic), 2962, 2911 (C—H aliphatic), 1697 (C=O), 1599, 1518 (C=C, C=N). ^1^H-NMR (δ, ppm): 8.35 (2H, *d*, *J =* 9.0 Hz, Ar-H), 8.21 (2H, *d*, *J =* 9.0 Hz, Ar-H), 7.99 (1H, *m*, Ar-H), 7.89 (1H, *m*, Ar-H), 7.76 (1H, *d*, *J* = 8.5 Hz, Ar-H), 7.48 (5H, *m*, Ar-H), 7.32 (2H, *dd*, *J_1_* = 7.5 Hz, *J_2_* = 1.5 Hz, Ar-H), 7.25 (1H, *t, J_1_* = *J_2_* = 7.5 Hz, Ar-H), 6.85 (1H, *d, J* = 7.0 Hz, Ar-H), 4.88 (2H, *s*, CH_2_), 4.43 (2H, *s*, –S—CH_2_—CO–). ^13^C-NMR (δ, ppm): 193.2 (C=O), 154.8, 150.6 (C=N), 150.0, 140.5, 133.7, 133.3, 132.0, 131.7, 130.5, 130.4, 130.3, 128.9, 127.9, 127.7, 127.4, 126.6, 126.2, 125.7, 124.3, 124.3 (C_Ar_), 39.4, 29.1 (–CH_2_–). HR-ESI-MS *m*/*z* 481.1325 (*M* + H)^+^ calculated for (C_27_H_20_N_4_O_3_S+H)^+^ 481.1334.

## 6. Refinement

Crystal data, data collection and structure refinement details are summarized in Table 2 ▸. All hydrogen atoms bound to carbon were placed at idealized positions and refined in riding mode, with *U*_iso_(H) values assigned as 1.2*U*_eq_ of the parent atoms, with C—H distances of 0.93 (aromatic) and 0.97 Å (CH_2_).

## Supplementary Material

Crystal structure: contains datablock(s) I. DOI: 10.1107/S2056989024000859/zv2033sup1.cif

Structure factors: contains datablock(s) I. DOI: 10.1107/S2056989024000859/zv2033Isup2.hkl

Refcodes CSD search. DOI: 10.1107/S2056989024000859/zv2033sup3.txt

Supporting information file. DOI: 10.1107/S2056989024000859/zv2033Isup4.cml

CCDC reference: 2327637

Additional supporting information:  crystallographic information; 3D view; checkCIF report

## Figures and Tables

**Table 1:** Selected interatomic distances (Å)

N2⋯H15^i^	2.69	O9⋯H7*A* ^iv^	2.61
S6⋯H24^ii^	2.91	O17⋯H23^v^	2.61
S6⋯O9^iii^	3.155 (3)	O18⋯H22^vi^	2.61

Symmetry codes: (i) 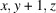 ; (ii) 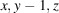 ; (iii) 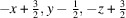 ; (iv) 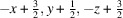 ; (v) 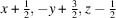 ; (vi) 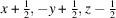 .

**Table 2:** Experimental details

Crystal data	
Chemical formula	C_27_H_20_N_4_O_3_S
*M*_r_	480.53
Crystal system, space group	Monoclinic, *P*2_1_/*n*
Temperature (K)	294
*a*, *b*, *c* (Å)	18.1825 (8), 5.6191 (3), 23.0548 (12)
β (°)	94.760 (4)
*V* (Å^3^)	2347.4 (2)
*Z*	4
Radiation type	Mo *K*α
μ (mm^−1^)	0.18
Crystal size (mm)	0.5 × 0.3 × 0.05

Data collection	
Diffractometer	SuperNova, Single source at offset/far, Eos
Absorption correction	Multi-scan (*CrysAlis PRO*; Rigaku OD, 2022 ▸)
*T*_min_, *T*_max_	0.683, 1.000
No. of measured, independent and observed [*I* > 2σ(*I*)] reflections	24984, 4768, 2795
*R*_int_	0.049
(sin θ/λ)_max_ (Å^−1^)	0.625

Refinement	
*R*[*F* ^2^ > 2σ(*F* ^2^)], *wR*(*F* ^2^), *S*	0.060, 0.153, 1.02
No. of reflections	4768
No. of parameters	316
H-atom treatment	H-atom parameters constrained
Δρ_max_, Δρ_min_ (e Å^−3^)	0.20, −0.20

Computer programs: *CrysAlis PRO* (Rigaku OD, 2022 ▸), *SHELXT2014/5* (Sheldrick, 2015*a* ▸), *SHELXL2016/4* (Sheldrick, 2015*b* ▸), and *OLEX2* (Dolomanov *et al.*, 2009 ▸).

## supplementary crystallographic information

### Crystal data

**Table:**

C_27_H_20_N_4_O_3_S	*F*(000) = 1000
*M**_r_* = 480.53	*D*_x_ = 1.360 Mg m^−^^3^
Monoclinic, *P*2_1_/*n*	Mo *K*α radiation, λ = 0.71073 Å
*a* = 18.1825 (8) Å	Cell parameters from 4787 reflections
*b* = 5.6191 (3) Å	θ = 3.0–23.2°
*c* = 23.0548 (12) Å	µ = 0.18 mm^−^^1^
β = 94.760 (4)°	*T* = 294 K
*V* = 2347.4 (2) Å^3^	Plate, yellow
*Z* = 4	0.5 × 0.3 × 0.05 mm

### Data collection

**Table:**

SuperNova, Single source at offset/far, Eos diffractometer	4768 independent reflections
Radiation source: micro-focus sealed X-ray tube, SuperNova (Mo) X-ray Source	2795 reflections with *I* > 2σ(*I*)
Mirror monochromator	*R*_int_ = 0.049
Detector resolution: 15.9631 pixels mm^-1^	θ_max_ = 26.4°, θ_min_ = 2.8°
ω scans	*h* = −22→22
Absorption correction: multi-scan (CrysAlisPro; Rigaku OD, 2022)	*k* = −7→7
*T*_min_ = 0.683, *T*_max_ = 1.000	*l* = −28→28
24984 measured reflections	

### Refinement

**Table:**

Refinement on *F*^2^	Primary atom site location: dual
Least-squares matrix: full	Hydrogen site location: inferred from neighbouring sites
*R*[*F*^2^ > 2σ(*F*^2^)] = 0.060	H-atom parameters constrained
*wR*(*F*^2^) = 0.153	*w* = 1/[σ^2^(*F*_o_^2^) + (0.0399*P*)^2^ + 1.5719*P*] where *P* = (*F*_o_^2^ + 2*F*_c_^2^)/3
*S* = 1.02	(Δ/σ)_max_ < 0.001
4768 reflections	Δρ_max_ = 0.20 e Å^−^^3^
316 parameters	Δρ_min_ = −0.20 e Å^−^^3^
0 restraints	

### Special details

**Table:**

Geometry. All esds (except the esd in the dihedral angle between two l.s. planes) are estimated using the full covariance matrix. The cell esds are taken into account individually in the estimation of esds in distances, angles and torsion angles; correlations between esds in cell parameters are only used when they are defined by crystal symmetry. An approximate (isotropic) treatment of cell esds is used for estimating esds involving l.s. planes.

### Fractional atomic coordinates and isotropic or equivalent isotropic displacement parameters (Å^2^)

**Table:**

	*x*	*y*	*z*	*U*_iso_*/*U*_eq_	
N1	0.57186 (16)	0.9491 (5)	0.59782 (12)	0.0701 (8)	
N2	0.62360 (14)	0.7920 (5)	0.62483 (11)	0.0651 (7)	
C3	0.60265 (15)	0.7508 (5)	0.67682 (13)	0.0534 (7)	
N4	0.53984 (13)	0.8750 (4)	0.68568 (10)	0.0527 (6)	
C5	0.52295 (17)	0.9964 (6)	0.63464 (14)	0.0605 (8)	
S6	0.64414 (4)	0.55907 (15)	0.72948 (3)	0.0593 (3)	
C7	0.69380 (15)	0.3774 (5)	0.68159 (13)	0.0571 (8)	
H7A	0.706046	0.227522	0.700906	0.068*	
H7B	0.661505	0.342471	0.647024	0.068*	
C8	0.76415 (16)	0.4880 (6)	0.66303 (13)	0.0555 (8)	
O9	0.79194 (12)	0.6603 (5)	0.68704 (10)	0.0818 (7)	
C10	0.79924 (15)	0.3730 (5)	0.61390 (12)	0.0531 (7)	
C11	0.85963 (17)	0.4841 (7)	0.59308 (15)	0.0765 (11)	
H11	0.876988	0.625964	0.609882	0.092*	
C12	0.89440 (19)	0.3871 (7)	0.54770 (16)	0.0820 (11)	
H12	0.935130	0.461787	0.533913	0.098*	
C13	0.86830 (18)	0.1820 (6)	0.52371 (14)	0.0663 (9)	
C14	0.8089 (2)	0.0679 (7)	0.54266 (17)	0.0886 (12)	
H14	0.791735	−0.072874	0.525179	0.106*	
C15	0.77436 (19)	0.1651 (6)	0.58847 (16)	0.0764 (10)	
H15	0.733878	0.088244	0.602056	0.092*	
N16	0.9043 (2)	0.0796 (7)	0.47416 (14)	0.0916 (10)	
O17	0.95542 (19)	0.1861 (6)	0.45607 (13)	0.1240 (12)	
O18	0.8816 (2)	−0.1084 (7)	0.45487 (16)	0.1493 (15)	
C19	0.50227 (15)	0.8822 (5)	0.73849 (13)	0.0550 (8)	
C20	0.4582 (2)	0.6984 (7)	0.75256 (19)	0.0946 (13)	
H20	0.451760	0.566655	0.728247	0.113*	
C21	0.4229 (2)	0.7105 (8)	0.8039 (2)	0.1094 (16)	
H21	0.392874	0.585700	0.814041	0.131*	
C22	0.4320 (2)	0.9020 (9)	0.83889 (19)	0.0945 (14)	
H22	0.407907	0.909348	0.872899	0.113*	
C23	0.4757 (2)	1.0827 (8)	0.82495 (16)	0.0903 (12)	
H23	0.481557	1.214769	0.849215	0.108*	
C24	0.51190 (18)	1.0725 (6)	0.77459 (14)	0.0699 (9)	
H24	0.542898	1.196205	0.765434	0.084*	
C25	0.45516 (19)	1.1441 (6)	0.62194 (16)	0.0765 (10)	
H25A	0.455159	1.208474	0.582903	0.092*	
H25B	0.456491	1.276913	0.648884	0.092*	
C26	0.38421 (19)	1.0048 (7)	0.62678 (17)	0.0763 (10)	
C27	0.36527 (18)	0.8164 (7)	0.58735 (16)	0.0713 (10)	
C28	0.4067 (2)	0.7616 (7)	0.53924 (15)	0.0756 (10)	
H28	0.447600	0.853610	0.532465	0.091*	
C29	0.3874 (2)	0.5778 (8)	0.50327 (18)	0.0909 (12)	
H29	0.415021	0.546766	0.471955	0.109*	
C30	0.3276 (3)	0.4357 (9)	0.5121 (2)	0.1076 (15)	
H30	0.315973	0.308241	0.487231	0.129*	
C31	0.2858 (2)	0.4807 (9)	0.5566 (2)	0.1062 (15)	
H31	0.245628	0.383158	0.562048	0.127*	
C32	0.3021 (2)	0.6746 (8)	0.59533 (19)	0.0860 (12)	
C33	0.2589 (2)	0.7287 (11)	0.6415 (2)	0.1214 (18)	
H33	0.217650	0.636414	0.647093	0.146*	
C34	0.2766 (3)	0.9150 (12)	0.6784 (2)	0.1254 (19)	
H34	0.247057	0.950357	0.708263	0.150*	
C35	0.3398 (2)	1.0526 (8)	0.67080 (19)	0.1007 (14)	
H35	0.351704	1.178344	0.696066	0.121*	

### Atomic displacement parameters (Å^2^)

**Table:**

	*U*^11^	*U*^22^	*U*^33^	*U*^12^	*U*^13^	*U*^23^
N1	0.0745 (18)	0.0761 (19)	0.0607 (17)	−0.0024 (16)	0.0113 (15)	0.0115 (15)
N2	0.0626 (16)	0.0795 (19)	0.0553 (16)	−0.0031 (15)	0.0165 (13)	0.0046 (14)
C3	0.0494 (17)	0.0586 (19)	0.0535 (18)	−0.0085 (15)	0.0127 (14)	0.0001 (14)
N4	0.0521 (14)	0.0531 (15)	0.0542 (15)	−0.0036 (12)	0.0125 (12)	0.0019 (12)
C5	0.062 (2)	0.0560 (19)	0.064 (2)	−0.0067 (16)	0.0062 (17)	0.0055 (16)
S6	0.0525 (5)	0.0748 (6)	0.0526 (5)	0.0014 (4)	0.0157 (4)	0.0037 (4)
C7	0.0510 (17)	0.0607 (19)	0.0610 (19)	−0.0017 (15)	0.0143 (14)	0.0013 (15)
C8	0.0481 (17)	0.065 (2)	0.0541 (18)	−0.0066 (15)	0.0090 (14)	−0.0035 (15)
O9	0.0672 (15)	0.0969 (18)	0.0843 (17)	−0.0281 (14)	0.0252 (13)	−0.0330 (15)
C10	0.0468 (16)	0.065 (2)	0.0482 (17)	−0.0035 (15)	0.0080 (13)	−0.0002 (14)
C11	0.059 (2)	0.097 (3)	0.076 (2)	−0.0258 (19)	0.0246 (18)	−0.023 (2)
C12	0.063 (2)	0.109 (3)	0.077 (2)	−0.022 (2)	0.0311 (19)	−0.011 (2)
C13	0.070 (2)	0.078 (2)	0.0533 (19)	0.0016 (19)	0.0205 (17)	−0.0011 (17)
C14	0.095 (3)	0.084 (3)	0.093 (3)	−0.023 (2)	0.043 (2)	−0.032 (2)
C15	0.075 (2)	0.072 (2)	0.088 (3)	−0.0183 (19)	0.038 (2)	−0.013 (2)
N16	0.100 (3)	0.107 (3)	0.074 (2)	0.000 (2)	0.0394 (19)	−0.011 (2)
O17	0.143 (3)	0.138 (3)	0.101 (2)	−0.012 (2)	0.078 (2)	−0.0025 (19)
O18	0.163 (3)	0.155 (3)	0.142 (3)	−0.033 (3)	0.081 (3)	−0.077 (3)
C19	0.0457 (16)	0.0598 (19)	0.0614 (19)	0.0028 (15)	0.0159 (14)	0.0072 (15)
C20	0.093 (3)	0.068 (2)	0.132 (4)	−0.020 (2)	0.058 (3)	−0.008 (2)
C21	0.096 (3)	0.091 (3)	0.151 (4)	−0.011 (3)	0.071 (3)	0.022 (3)
C22	0.082 (3)	0.116 (4)	0.091 (3)	0.031 (3)	0.043 (2)	0.034 (3)
C23	0.096 (3)	0.113 (3)	0.065 (2)	0.007 (3)	0.027 (2)	−0.009 (2)
C24	0.070 (2)	0.079 (2)	0.063 (2)	−0.0109 (18)	0.0183 (17)	−0.0015 (18)
C25	0.082 (2)	0.064 (2)	0.082 (3)	0.007 (2)	−0.002 (2)	0.0082 (19)
C26	0.064 (2)	0.083 (3)	0.081 (3)	0.015 (2)	0.000 (2)	0.013 (2)
C27	0.061 (2)	0.080 (3)	0.070 (2)	0.0061 (19)	−0.0075 (18)	0.022 (2)
C28	0.075 (2)	0.089 (3)	0.062 (2)	−0.006 (2)	−0.0051 (19)	0.015 (2)
C29	0.091 (3)	0.107 (3)	0.072 (3)	−0.016 (3)	−0.008 (2)	0.011 (2)
C30	0.110 (4)	0.120 (4)	0.088 (3)	−0.019 (3)	−0.021 (3)	0.007 (3)
C31	0.082 (3)	0.123 (4)	0.109 (4)	−0.033 (3)	−0.024 (3)	0.025 (3)
C32	0.057 (2)	0.115 (3)	0.084 (3)	−0.004 (2)	−0.005 (2)	0.026 (3)
C33	0.061 (3)	0.179 (6)	0.124 (4)	−0.008 (3)	0.006 (3)	0.029 (4)
C34	0.066 (3)	0.195 (6)	0.118 (4)	0.021 (3)	0.021 (3)	0.002 (4)
C35	0.076 (3)	0.124 (4)	0.104 (3)	0.029 (3)	0.015 (2)	−0.009 (3)

### Geometric parameters (Å, º)

**Table:**

N1—N2	1.398 (4)	C20—C21	1.394 (5)
N1—C5	1.307 (4)	C21—H21	0.9300
N2—C3	1.308 (3)	C21—C22	1.347 (6)
C3—N4	1.368 (3)	C22—H22	0.9300
C3—S6	1.747 (3)	C22—C23	1.345 (5)
N4—C5	1.373 (4)	C23—H23	0.9300
N4—C19	1.445 (3)	C23—C24	1.383 (4)
C5—C25	1.495 (4)	C24—H24	0.9300
S6—C7	1.800 (3)	C25—H25A	0.9700
C7—H7A	0.9700	C25—H25B	0.9700
C7—H7B	0.9700	C25—C26	1.521 (5)
C7—C8	1.515 (4)	C26—C27	1.419 (5)
C8—O9	1.205 (3)	C26—C35	1.375 (5)
C8—C10	1.492 (4)	C27—C28	1.425 (5)
C10—C11	1.383 (4)	C27—C32	1.423 (5)
C10—C15	1.367 (4)	C28—H28	0.9300
C11—H11	0.9300	C28—C29	1.352 (5)
C11—C12	1.379 (4)	C29—H29	0.9300
C12—H12	0.9300	C29—C30	1.377 (5)
C12—C13	1.347 (5)	C30—H30	0.9300
C13—C14	1.359 (4)	C30—C31	1.351 (6)
C13—N16	1.480 (4)	C31—H31	0.9300
C14—H14	0.9300	C31—C32	1.424 (6)
C14—C15	1.385 (4)	C32—C33	1.407 (6)
C15—H15	0.9300	C33—H33	0.9300
N16—O17	1.208 (4)	C33—C34	1.370 (7)
N16—O18	1.206 (4)	C34—H34	0.9300
C19—C20	1.363 (4)	C34—C35	1.407 (6)
C19—C24	1.358 (4)	C35—H35	0.9300
C20—H20	0.9300		
			
N2···H15^i^	2.69	O9···H7A^iv^	2.61
S6···H24^ii^	2.91	O17···H23^v^	2.61
S6···O9^iii^	3.155 (3)	O18···H22^vi^	2.61
			
C5—N1—N2	108.0 (3)	C20—C21—H21	119.8
C3—N2—N1	106.5 (2)	C22—C21—C20	120.3 (4)
N2—C3—N4	110.9 (3)	C22—C21—H21	119.8
N2—C3—S6	127.2 (2)	C21—C22—H22	119.8
N4—C3—S6	121.9 (2)	C23—C22—C21	120.4 (4)
C3—N4—C5	104.8 (2)	C23—C22—H22	119.8
C3—N4—C19	126.8 (2)	C22—C23—H23	120.0
C5—N4—C19	128.3 (3)	C22—C23—C24	120.1 (4)
N1—C5—N4	109.9 (3)	C24—C23—H23	120.0
N1—C5—C25	125.5 (3)	C19—C24—C23	120.1 (3)
N4—C5—C25	124.5 (3)	C19—C24—H24	120.0
C3—S6—C7	97.64 (14)	C23—C24—H24	120.0
S6—C7—H7A	108.6	C5—C25—H25A	109.0
S6—C7—H7B	108.6	C5—C25—H25B	109.0
H7A—C7—H7B	107.6	C5—C25—C26	113.0 (3)
C8—C7—S6	114.8 (2)	H25A—C25—H25B	107.8
C8—C7—H7A	108.6	C26—C25—H25A	109.0
C8—C7—H7B	108.6	C26—C25—H25B	109.0
O9—C8—C7	122.1 (3)	C27—C26—C25	119.9 (3)
O9—C8—C10	120.4 (3)	C35—C26—C25	120.5 (4)
C10—C8—C7	117.5 (3)	C35—C26—C27	119.5 (4)
C11—C10—C8	118.0 (3)	C26—C27—C28	123.0 (3)
C15—C10—C8	123.2 (3)	C26—C27—C32	119.4 (4)
C15—C10—C11	118.7 (3)	C32—C27—C28	117.7 (4)
C10—C11—H11	119.6	C27—C28—H28	119.5
C12—C11—C10	120.8 (3)	C29—C28—C27	121.0 (4)
C12—C11—H11	119.6	C29—C28—H28	119.5
C11—C12—H12	120.6	C28—C29—H29	119.3
C13—C12—C11	118.8 (3)	C28—C29—C30	121.4 (4)
C13—C12—H12	120.6	C30—C29—H29	119.3
C12—C13—C14	122.3 (3)	C29—C30—H30	119.9
C12—C13—N16	119.1 (3)	C31—C30—C29	120.3 (5)
C14—C13—N16	118.6 (3)	C31—C30—H30	119.9
C13—C14—H14	120.6	C30—C31—H31	119.4
C13—C14—C15	118.8 (3)	C30—C31—C32	121.3 (4)
C15—C14—H14	120.6	C32—C31—H31	119.4
C10—C15—C14	120.6 (3)	C27—C32—C31	118.4 (4)
C10—C15—H15	119.7	C33—C32—C27	118.9 (5)
C14—C15—H15	119.7	C33—C32—C31	122.7 (5)
O17—N16—C13	118.4 (4)	C32—C33—H33	119.4
O18—N16—C13	117.7 (3)	C34—C33—C32	121.2 (5)
O18—N16—O17	123.8 (4)	C34—C33—H33	119.4
C20—C19—N4	120.5 (3)	C33—C34—H34	120.2
C24—C19—N4	119.5 (3)	C33—C34—C35	119.7 (5)
C24—C19—C20	120.0 (3)	C35—C34—H34	120.2
C19—C20—H20	120.4	C26—C35—C34	121.3 (5)
C19—C20—C21	119.1 (4)	C26—C35—H35	119.3
C21—C20—H20	120.4	C34—C35—H35	119.3
			
N1—N2—C3—N4	−0.6 (3)	C12—C13—N16—O17	−1.3 (6)
N1—N2—C3—S6	177.5 (2)	C12—C13—N16—O18	177.3 (4)
N1—C5—C25—C26	117.2 (4)	C13—C14—C15—C10	0.5 (6)
N2—N1—C5—N4	−0.3 (4)	C14—C13—N16—O17	177.3 (4)
N2—N1—C5—C25	−175.6 (3)	C14—C13—N16—O18	−4.0 (6)
N2—C3—N4—C5	0.4 (3)	C15—C10—C11—C12	−0.3 (5)
N2—C3—N4—C19	−177.2 (3)	N16—C13—C14—C15	−179.0 (4)
N2—C3—S6—C7	−20.3 (3)	C19—N4—C5—N1	177.5 (3)
C3—N4—C5—N1	0.0 (3)	C19—N4—C5—C25	−7.1 (5)
C3—N4—C5—C25	175.3 (3)	C19—C20—C21—C22	0.3 (7)
C3—N4—C19—C20	−78.3 (4)	C20—C19—C24—C23	−1.5 (5)
C3—N4—C19—C24	100.9 (4)	C20—C21—C22—C23	−0.5 (7)
C3—S6—C7—C8	78.5 (2)	C21—C22—C23—C24	−0.3 (7)
N4—C3—S6—C7	157.6 (2)	C22—C23—C24—C19	1.3 (6)
N4—C5—C25—C26	−57.4 (4)	C24—C19—C20—C21	0.7 (6)
N4—C19—C20—C21	179.8 (4)	C25—C26—C27—C28	−5.6 (5)
N4—C19—C24—C23	179.3 (3)	C25—C26—C27—C32	174.7 (3)
C5—N1—N2—C3	0.5 (3)	C25—C26—C35—C34	−175.6 (4)
C5—N4—C19—C20	104.7 (4)	C26—C27—C28—C29	178.9 (3)
C5—N4—C19—C24	−76.1 (4)	C26—C27—C32—C31	−177.6 (3)
C5—C25—C26—C27	−65.5 (4)	C26—C27—C32—C33	1.5 (5)
C5—C25—C26—C35	111.5 (4)	C27—C26—C35—C34	1.4 (6)
S6—C3—N4—C5	−177.8 (2)	C27—C28—C29—C30	−0.6 (6)
S6—C3—N4—C19	4.6 (4)	C27—C32—C33—C34	0.3 (7)
S6—C7—C8—O9	13.8 (4)	C28—C27—C32—C31	2.7 (5)
S6—C7—C8—C10	−167.1 (2)	C28—C27—C32—C33	−178.2 (4)
C7—C8—C10—C11	174.3 (3)	C28—C29—C30—C31	1.3 (7)
C7—C8—C10—C15	−5.3 (5)	C29—C30—C31—C32	0.1 (7)
C8—C10—C11—C12	−179.9 (3)	C30—C31—C32—C27	−2.1 (6)
C8—C10—C15—C14	179.5 (3)	C30—C31—C32—C33	178.9 (4)
O9—C8—C10—C11	−6.6 (5)	C31—C32—C33—C34	179.3 (4)
O9—C8—C10—C15	173.8 (3)	C32—C27—C28—C29	−1.4 (5)
C10—C11—C12—C13	0.3 (6)	C32—C33—C34—C35	−1.2 (8)
C11—C10—C15—C14	−0.1 (5)	C33—C34—C35—C26	0.3 (7)
C11—C12—C13—C14	0.1 (6)	C35—C26—C27—C28	177.4 (3)
C11—C12—C13—N16	178.6 (3)	C35—C26—C27—C32	−2.3 (5)
C12—C13—C14—C15	−0.5 (6)		

Symmetry codes: (i) *x*, *y*+1, *z*; (ii) *x*, *y*−1, *z*; (iii) −*x*+3/2, *y*−1/2, −*z*+3/2; (iv) −*x*+3/2, *y*+1/2, −*z*+3/2; (v) *x*+1/2, −*y*+3/2, *z*−1/2; (vi) *x*+1/2, −*y*+1/2, *z*−1/2.
